# Influence of Age on Patellar Tendon Reflex Response

**DOI:** 10.1371/journal.pone.0080799

**Published:** 2013-11-18

**Authors:** Annapoorna Chandrasekhar, Noor Azuan Abu Osman, Lai Kuan Tham, Kheng Seang Lim, Wan Abu Bakar Wan Abas

**Affiliations:** 1 Department of Biomedical Engineering, Faculty of Engineering, University of Malaya, Kuala Lumpur, Malaysia; 2 Division of Neurology, Faculty of Medicine, University of Malaya, Kuala Lumpur, Malaysia; Universidad Europea de Madrid, Spain

## Abstract

**Background:**

A clinical parameter commonly used to assess the neurological status of an individual is the tendon reflex response. However, the clinical method of evaluation often leads to subjective conclusions that may differ between examiners. Moreover, attempts to quantify the reflex response, especially in older age groups, have produced inconsistent results. This study aims to examine the influence of age on the magnitude of the patellar tendon reflex response.

**Methodology/Principal Findings:**

This study was conducted using the motion analysis technique with the reflex responses measured in terms of knee angles. Forty healthy subjects were selected and categorized into three different age groups. Patellar reflexes were elicited from both the left and right patellar tendons of each subject at three different tapping angles and using the Jendrassik maneuver. The findings suggested that age has a significant effect on the magnitude of the reflex response. An angle of 45° may be the ideal tapping angle at which the reflex can be elicited to detect age-related differences in reflex response. The reflex responses were also not influenced by gender and were observed to be fairly symmetrical.

**Conclusions/Significance:**

Neurologically normal individuals will experience an age-dependent decline in patellar reflex response.

## Introduction

The tendon jerk, one of the most frequently used clinical methods for the differential assessment of human motor disorders [1]–[5], is classified as a type of somatic reflex [6]. Such reflexes can be observed by tapping the tendon of a muscle, and they are most distinct at the patellar tendon of the quadriceps femoris and the triceps tendon above the elbow [6]. The present study focuses on the reflex of the patellar tendon, which is categorized as a deep tendon, the monosynaptic reflex [7], and the variation of the response with age.

Generally, as an individual ages, the neuromuscular system is reported to be affected by structural and functional changes that tend to lead to a gradual decline in neuromuscular performance [8]–[10]. However, much of our understanding regarding the effect of age on the magnitude of the reflex response remains speculative given that most studies that have analyzed the effect of age on reflex responses have produced inconsistent results [10], [11]. Some studies [10]–[14] reported that the elderly have weak and delayed neuromuscular reflexes, whereas others have reported no change [15]–[18] or even enhanced amplitude [19] in reflex response with ageing [10]. These inconsistencies may be due to a lack of standardized, accurate, and convenient methods to quantify tendon reflex response in large samples while minimizing other extraneous variables [10]. The amplitude of the tendon jerk is clinically evaluated on a four- or five-point scale [1], [20], [21]. The problem with the clinical method of evaluation is that a certain degree of ambiguity remains in the physiological interpretation of the test, especially in evaluating the functional status of the patient [4], [20]. Many researchers have attempted several other means of quantifying reflex response in an effort to increase the objectivity and accuracy of the assessment [11], [22]. Some of the techniques include surface electromyography [9], [23]–[26], imaging techniques (MRI, ultrasound) [1], and the development of instrumented reflex hammers [24]–[28]. However, the drawback of these techniques is that they are often cumbersome, involve extensive analysis, increase reflex variability or could cause discomfort, thereby hindering clinical implementation [22]. Thus, alternative forms of assessment that are quantitative and accurate, yet convenient, comprehensive and clinically relevant, are still lacking [10]. Motion analysis is an approach that has been suggested recently for the assessment of patellar tendon reflex response, and it has proven to be a valid and reliable technique that could possibly overcome the limitations of the current methods [4], [22].

This study uses the same technique to quantify reflex response, but with notable differences, to further investigate patellar tendon reflex response. Key differences include: first, the focus of this study is on the influence of age on the magnitude of the reflex response, and second, unlike many studies [8]–[11], [16] that have previously investigated the effects of ageing by performing a stark contrast comparison between young adults and the elderly, this study aims to investigate the reflex response among three age groups with a gradual change in age. We believe that this technique can provide a better understanding of the variations in reflex response that occur with increase in age.

## Materials and Methods

The study protocols have been approved by a review board at the Department of Biomedical Engineering at the University of Malaya, Malaysia.

A reflex quantification device was built to firmly hold the reflex hammer at the desired position. The holder consists of a vertical beam and a horizontal rod, to which a Queen's reflex hammer and set-square were attached. A screw was used to fix the set-square to the tip of the reflex hammer to measure the tapping angle, and fasten the entire structure onto the horizontal rod. This helps to restrict the motion of the reflex hammer to a single plane [4]. The horizontal rod could then slide across the length of the vertical beam to adjust the position of the reflex hammer to the height of the subject's patellar tendon. The threaded portion of the horizontal rod functions to clamp the reflex hammer with the aid of a bolt and nut. This device is similar to the model developed in an earlier study [4].

A total of 40 subjects, 19 males and 21 females, participated in the study. The participants were assigned to three different groups on the basis of age: Group 1 (26–38 years old), Group 2 (39–51 years old), and Group 3 (52–64 years old). Table 1 shows their anthropometric information. All subjects selected were healthy volunteers, and the participants were recruited by opportunity sampling [21]. All subjects were unfamiliar to the experimental method and had not participated in any similar research. Each subject was asked to fill in a form to provide background health information and to check for previous or known existent neurological conditions or diseases.

**Table 1:** Anthropometric data of the subjects.

Age group	1	2	3
N	14 (M = 7; F = 7)	14 (M = 6; F = 8)	12 (M = 6; F = 6)
Height (m)	1.66±0.09	1.61±0.10	1.60±0.11
Body mass (kg)	64.96±13.01	68.14±12.63	62.6±9.95

Individuals under the age of 25 years and those who had any history of neurological disorders were excluded from the study. Subjects gave their written informed consent and were free to withdraw from the study at any point. All participants were compensated for their time and effort.

**Figure 1:**
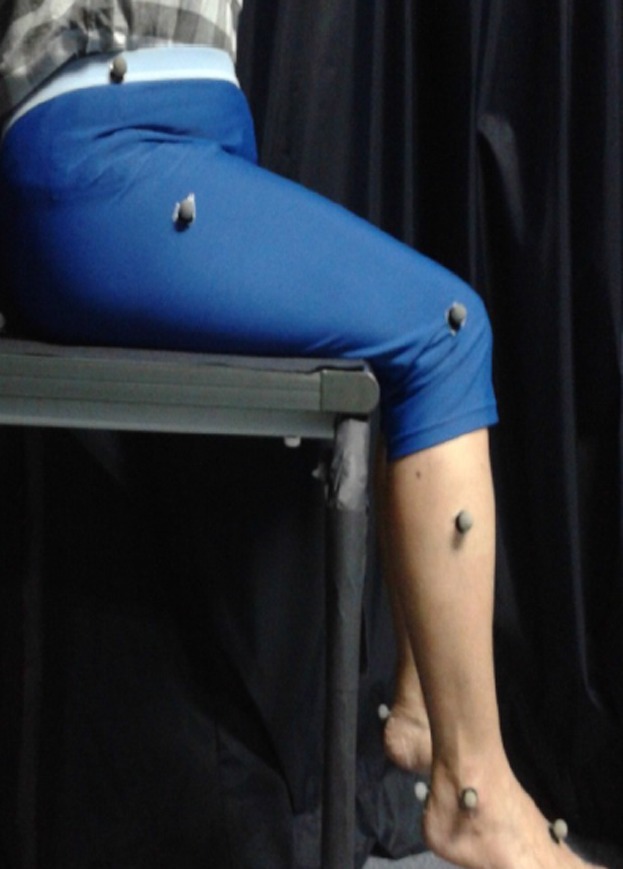
Experimental set-up.

**Figure 2:**
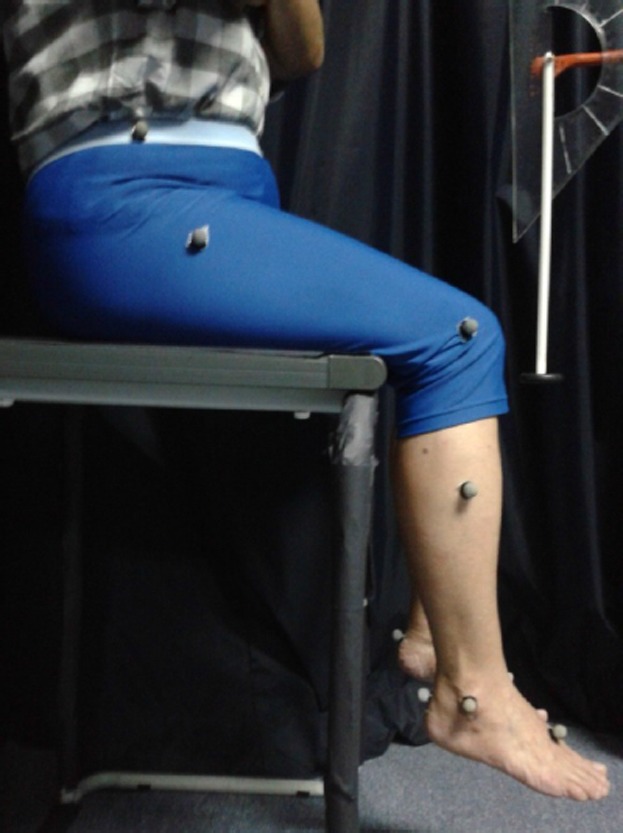
Position during reflex testing.

Sixteen reflective markers were attached to the subject's lower limbs following the Plug-in Gait Marker Placement guide [22]. A high platform was prepared such that subjects could be seated on top, with the knees slightly flexed, in a manner by which both the legs dangled freely without touching the ground [23]. The limbs were ensured to be in a relaxed and symmetric position, as it could influence the reflex amplitude. The experimental setup is shown in Figure 1. The tendon taps were administered manually with the head of the Queen's reflex hammer, the orientation of which was ensured to be at the same level as the tendon before being released from a particular angle measured by the attached set-square [22], [23], as shown in Figure 2.

To maximize the amplitude and success rate of the reflex response, the most sensitive region that elicits the reflex was located and marked [23] through a few pre-trial tendon taps performed at random time intervals (1-10 s). This spot on the right patellar tendon was then tapped five times with the Queen's square reflex hammer, released at an angle of 45°, with no external force being exerted on the hammer. The same procedure was repeated at two other angles (60° and 90°) as well as the Jendrassik maneuver. The tapping force was derived from the potential energy of the reflex hammer, which in turn depends on the angle to which the reflex hammer is raised. All tests were then repeated on the left patellar tendon. The reflex response was allowed to stop naturally, and the next tap was applied only when the lower leg was stationary. A minimum rest period of at least 5 s was provided in between taps [23]. This procedure was repeated by a qualified medical physician in the same manner on both legs of all the subjects.

For the Jendrassik maneuver (performed at 90°), the subjects were instructed to interlock their left and right hands and pull in response to an oral prompt given just about 2 s before tapping the tendon to create maintained tension across the shoulders. A second command to terminate this maneuver followed [23]. The tapping process was recorded by the motion analysis system Vicon Nexus 1.8.1, where a playback of the experimental video allows for a detailed analysis. Subjects were given adequate time between taps to rest and the option to request that we discontinue the experiment if they were experiencing any discomfort.

A statistical analysis of the data was carried out using the SPSS statistical software for Windows (V 20; IBM Corp., Armonk, NY) [22]. Comparisons between the different age groups, genders, and also the left and right patellar tendon reflex responses were performed. The joint mean angles were compared between genders and the left and right sides of the body using independent t-tests with a statistical significance of p<0.05 [4]. The effect of age on the magnitude of patellar tendon reflex response was analyzed using one way analysis of variance (ANOVA), and Tukey's HSD test was used as the post hoc analysis with p<0.05 for multiple-pair comparisons among the three age groups [4]. All data obtained were represented as mean±standard deviation, and the results were considered as statistically significant when p<0.05.

## Results

**Figure 3:**
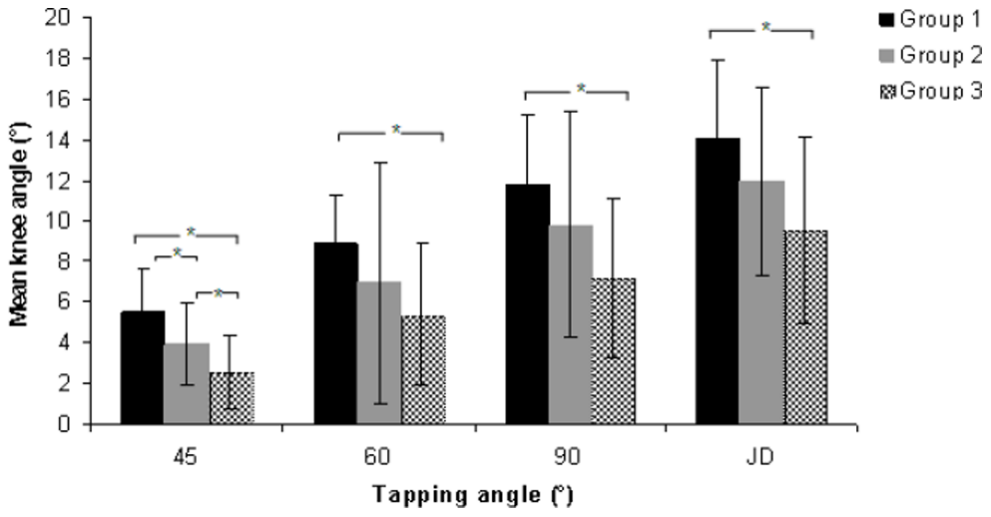
Comparison of reflex response among the three age groups at different tapping angles.

**Figure 4:**
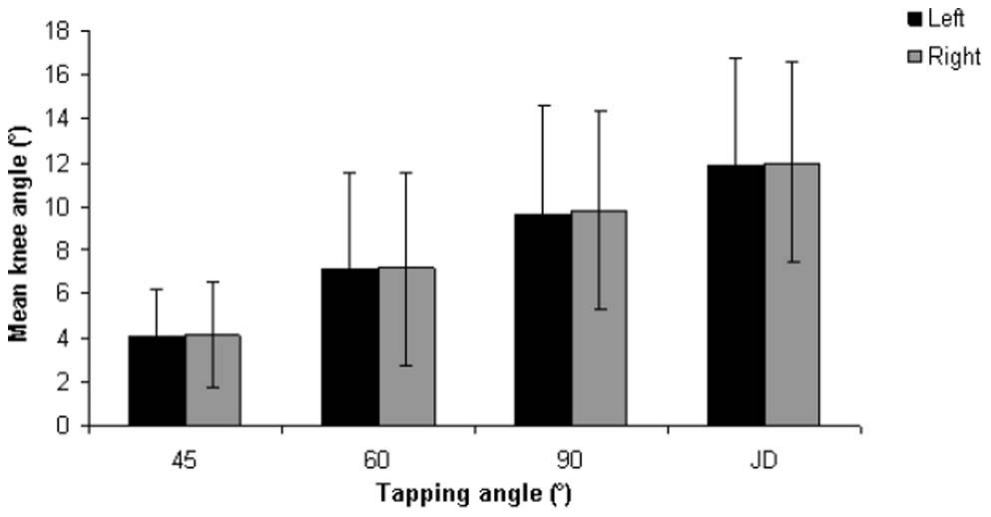
Comparison of reflex response between the right and left patellar tendons.

**Figure 5:**
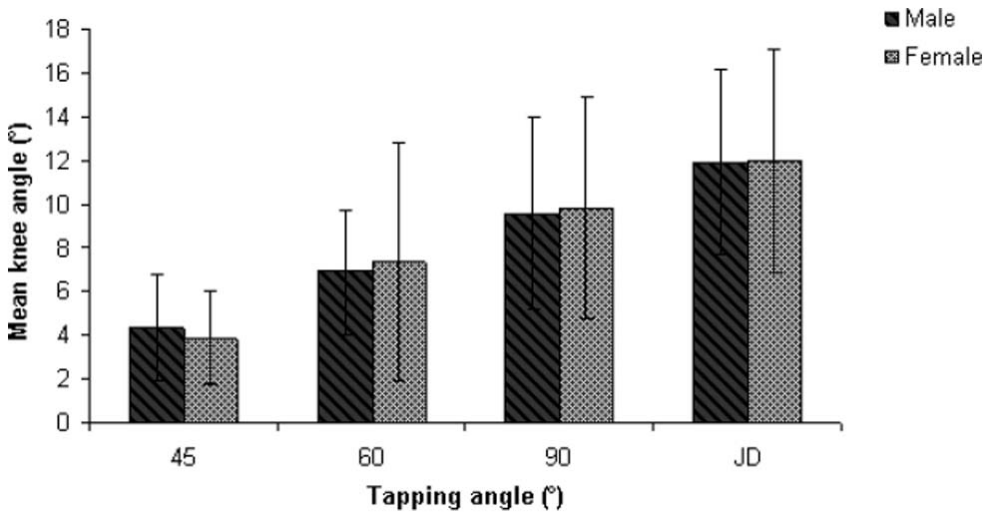
Comparison of reflex response between males and females.

The mean reflex response obtained at different tapping angles for the three different age groups are summarized in Table 2. The reflex responses were significantly different at all tapping angles and with the reinforcement technique. The trends observed include a decline in reflex response with an increase in age and an increase in the magnitude of reflex response with larger tapping angles (Figure 3). Additional post-hoc tests revealed that the significant differences were predominant between Group 1 and 3. However, at the tapping angle of 45°, significant differences were noted among all three age groups (Figure 3). The mean reflex responses obtained at the left and right patellar tendon are tabulated in Table 3. Overall, no significant difference was found in the reflex response between the left and right side, although reflex responses were slightly higher on the right side (Figure 4). Table 4 shows the mean values of the reflex response obtained in males and females at different tapping angles. The independent t-test indicates no significant difference in reflex response between males and females at all tapping angles. Female subjects generally had slightly higher responses compared to males, although the differences were not significant (Figure 5).

**Table 2:** Comparison of the reflex response amongst the three age groups at different tapping angles.

Tapping angle (°)	Age group	N	Mean angle (°)	*P* value
45	1	28	5.51±2.11	<0.001*
	2	28	3.93±1.99	
	3	24	2.59±1.80	
60	1	28	8.83±2.44	0.016*
	2	28	6.94±5.91	
	3	24	5.38±3.46	
90	1	28	11.82±3.42	0.001*
	2	28	9.82±5.54	
	3	24	7.18±3.91	
JD	1	28	14.04±3.90	0.002*
	2	28	11.93±4.68	
	3	24	9.54±4.59	

JD: Jendrassik maneuver.

* Statistically significant differences (*P*<0.05).

**Table 3:** Mean knee angles for the comparison of the reflex response between right and left lower limb.

Tapping angle (°)	Mean knee angle (°)		*P* value
	Right (N = 40)	Left (N = 40)	
45	4.13±2.41	4.04±2.19	0.862
60	7.16±4.41	7.10±4.46	0.948
90	9.83±4.57	9.62±4.96	0.845
JD	12.01±4.55	11.89±4.91	0.905

JD: Jendrassik maneuver.

**Table 4:** Mean knee angles for the comparison of the reflex response between males and females.

Tapping angle (°)	Mean knee angle (°)		*P* value
	Male (N = 38)	Female (N = 42)	
45	4.35±2.44	3.85±2.14	0.331
60	6.88±2.91	7.36±5.45	0.616
90	9.58±4.40	9.86±5.08	0.792
JD	11.93±4.25	11.97±5.13	0.963

JD: Jendrassik maneuver.

## Discussion

Two different angles are involved in this study: First is the tapping angle, referring to the reflex hammer's release angle; second is the knee angle, which serves as a measurement of the reflex amplitude in response to tapping angle.

The increase we observed in the reflex response at larger tapping angles and the results we obtained for the youngest age group (Group 1) were found to be in accordance with the findings of a study [22] conducted for a similar age group. In the present study, the patellar tendon reflex response of the oldest subjects (Group 3) differed from the groups of younger ages (Group 1 and 2). Although one-way ANOVA revealed a significant difference among all the age groups for all tapping angles, additional post-hoc tests confirmed that the significant differences in reflex response were mainly between Group 1 (26–38 years old) and Group 3 (52–64 years old). In contrast to the results at the 45° tapping angle, all the other tapping angles and the reinforcement technique did not show significant differences between Group 1 and 2, or between Group 2 and 3. These results were found to be consistent with the findings of previous researchers, who suggested that the decline in the reflex response becomes distinct after the age of 50 [29]. The findings of this study were also found to be in agreement with the reports that investigated the effect of age on reflex responses by comparing the reflexes of young adults and elderly individuals [8], [10], [12], [14]. Although this result suggests that differences do not exist in the middle range (Group 2), this may not be the case considering the limited population used in this study. A different pattern was observed at the 45° tapping angle as well.

At the 45° tapping angle, significant differences were noted among all three age groups. Moreover, Tham et al. (2013) also recommended either 45° or 60° as the optimal tapping angle to elicit the patellar reflex. Thus, the findings of this study support the 45° angle as the ideal tapping angle to elicit the reflex response to detect age-related differences. The findings of this study are in disagreement with previous research suggesting that age does not affect [15]–[17] or enhance reflex response [19]. The difference in outcomes may be due to the fact that those studies did not implement adequate controls [10], imposed gender restrictions [15] or were conducted several years back [15]–[17], [19]. Moreover, the previous studies lacked accurate and standardized yet convenient methods to quantify the reflex response for large-sized populations [10], which is possible with the motion analysis technique [22].

Two possible mechanisms have been suggested as causes for the decline in the reflex response of older subjects. First, the observed decline could be due to the weaker muscle contractions that are a result of the progressive decrease in the number of muscle fibers as individuals grow older [29]. Ageing is often associated with a loss of muscle mass that leads to a decrease in muscle force and power [29]. The study conducted by Kim et al. (2010) showed a significant degradation in knee and ankle muscle strength with an increase in age. Younger adults had stronger muscle strength compared with the elderly, whereas middle-age adults did not have statistically different muscle strength compared with the elderly subjects [30]. This finding indicated that the attenuation of muscle strength is similar in both the middle-age adults and the elderly when compared with the younger adult group [30]. Second, reduced neuronal excitations may be involved [8], [10], but age-related deteriorations of the reflex response are reported to be more dominantly influenced by changes in the contractile properties of the muscle [10]. Further research is required to develop an understanding of the fundamental causes of age-related changes that would help to distinguish whether they are of neuronal or muscular origin. [10]

In this study, the physical activity or fitness levels of participants were not quantified. Recent research suggests that ageing might actually affect muscle function indirectly [21]. Inactivity and atrophy that is associated with ageing might actually be the cause of the reduction in reflex responses rather than age itself [21]. Further studies are required to investigate this hypothesis. However, it is believed that this study adds to the evidence base, elucidates an area for further research that may be important, and has clinical relevance [21].

The standard deviations in the results are not unusual considering that studies suggest the wide fluctuations in human tendon reflexes among the healthy population [3] even with the use of accurate instruments for stimulation [31]. The absence of reflexes was also noted in two individuals of the oldest age group (52–64 years old) in this study, which is reportedly a common phenomenon observed among elderly subjects [3], [5].

Based on the results presented in Table 4, reflex responses were generally higher among female subjects than in males, although the difference is not statistically significant. Very few studies have actually focused on gender differentiation as their primary objective, and their findings are difficult to compare because different researchers often use different parameters to quantify the reflex response [23]. In a study [23] that compared the differences in reflex response between males and females using surface electromyography, the findings suggest that males have a slower patellar reflex compared with females. A slower conduction velocity and a larger cross-sectional area of spinal motor neurons are present in males than females [23], which could explain the difference. Similarly, Chung et al. (2005) and Tham et al. (2011) reported higher but non-significant reflex responses among females. They suggest that this result is due to the greater sensitivity of female subjects to the tendon taps administered during the experimental trials [4], [10]. Some studies have reported conflicting results [32]. However, in this study, considering that there were no significant differences observed at all tapping angles and the results were found to be in agreement with several recently published works, gender was not expected to significantly influence reflex responses.

Asymmetrical reflex responses indicate the presence of abnormalities. Thus, these responses should not usually be observed among the healthy population [3], [4]. Neurological disorders are often diagnosed based on the presence of an asymmetrical reflex response [3]. However, non-significant differences could exist, as subjects normally tend to have a stronger response on their dominant side [4]. The results shown in Table 3 and Figure 4 suggest that reflex responses tend to be slightly higher on the right side. Most of the subjects who participated in this study have a dominant right side; therefore, this result is expected.

The Jendrassik maneuver is a reinforcement technique that is used to bring out the reflex in patients who are having difficulty relaxing [2]. This maneuver isometrically activates the muscles in the upper extremities [2]. This technique was observed to elicit greater reflex responses than those elicited normally without reinforcement (Tables 2, 3, and 4). Several possible theories have been proposed for the ability of this maneuver to bring out a greater reflex response compared with non-reinforcement techniques, but we are still uncertain of the exact mechanism that causes this effect [4], [11].

The results of this study were found to be significant as they helped quantify the relationship between age and reflex response using an accurate method with easily relatable parameters. The ambiguities commonly encountered in a clinical setting could be avoided with the implementation of this technique to quantify reflex response [22]. We believe that this study can contribute to the development of a standardized database with normative data for healthy subjects, classified according to age, height, gender, and so on. This database would be a novel resource that would enable researchers and medical practitioners to estimate and predict the range of reflex response for a healthy individual of a particular age. This would help to differentiate normal subjects from those with abnormalities and aid in the design of improved rehabilitation interventions for elderly individuals [10].

The present assessment is confined to the bounds of an experimental research laboratory setting equipped with motion analysis systems such as the Vicon Nexus. However, with further progress in this field, a potential exists for the development of portable systems enabling the performance of this assessment in the clinical setting [4], [22]. Future work that could be conducted includes performing this experiment on a larger scale, which would increase the accuracy of the data collected and help increase confidence in this form of testing. Latency is another factor that could be explored to further analyze the effect of age [10], [23]. This form of testing could also be used in experiments on subjects with neurological disorders to determine the quantitative extent of reflex response variation and compare it with the set of normative data to develop a greater understanding of the reflex testing procedure's significance.

## Conclusion

The major finding of this study confirms a decline in the magnitude of reflex response with increasing age. The differences can be best observed at a tapping angle of 45°. This result suggests that age is an important factor that influences the reflex response of an individual, and the extent to which the reflex response is affected has been quantified in terms of knee angles. This study also suggests that gender and limb position (right/left side) do not have a significant impact on the magnitude of reflex response. The findings of this study are believed to have the potential to contribute to the development of an evidence base that could help physicians perform more accurate reflex assessments.
